# Supplementary material for: The impact of heating, ventilation and air conditioning (HVAC) design features on the transmission of viruses, including the 2019 novel coronavirus (COVID-19): A systematic review of humidity
Source: PLoS One. 2022 Oct 10;17(10):e0275654. doi: 10.1371/journal.pone.0275654 (PMC9550073; doi:10.1371/journal.pone.0275654)
Supplement: S1 File — (DOCX) [file pone.0275654.s002.docx]

**S1 Appendix A: Search Strategy for Ovid MEDLINE^21^**

Database: Ovid MEDLINE(R) ALL 1946 to Present

Search Strategy:

| **#** | **Searches** |
| --- | --- |
| 1 | exp Aerosols/ |
| 2 | Air Microbiology/ |
| 3 | exp Viruses/ |
| 4 | (aerosol or aerosols or bioaerosol or bioaerosols).mp. |
| 5 | droplet nuclei.mp. |
| 6 | infectio*.mp. |
| 7 | (pathogen or pathogens).mp. |
| 8 | (virus or viruses or viral or virome).mp. |
| 9 | or/1-8 [MeSH + Keywords – Virus concept] |
| 10 | Air Conditioning/ |
| 11 | Air Filters/ or Filtration/ |
| 12 | Humidity/ |
| 13 | Ventilation/ |
| 14 | Ultraviolet Rays/ |
| 15 | air condition*.mp. |
| 16 | (air change rate or air change rates or air changes per hour or air exchange rate or air exchange rates or air exchanges per hour).mp. |
| 17 | (airflow or air flow).mp. |
| 18 | built environment.mp. |
| 19 | computational fluid dynamics.mp. |
| 20 | ((distance adj6 index) or long distances).mp. |
| 21 | HVAC.mp. |
| 22 | (filter or filters or filtration).mp. |
| 23 | humidity.mp. |
| 24 | (ultraviolet or UV).mp. |
| 25 | ventilat*.mp. |
| 26 | or/10-25 [MeSH + Keywords – HVAC concept] |
| 27 | Air Pollution, Indoor/ |
| 28 | exp Disease Transmission, Infectious/ |
| 29 | (indoor adj1 (air quality or environment*)).mp. |
| 30 | transmission.mp. |
| 31 | or/27-30 [MeSH + Keywords – Transmission concept] |
| 32 | 9 and 26 and 31 |
| 33 | remove duplicates from 32 |

MeSH = Medical Subject Headings

**S1 Appendix B. Inclusion and exclusion criteria for systematic review^21^**

| **Item** | **Inclusion criteria** | **Exclusion criteria** |
| --- | --- | --- |
| Agent | - Viruses - Aerosols - Bioaerosols - Droplet nuclei - Other pathogens (e.g., bacteria, fungi)   *We planned a staged process: if we identified studies specific to viruses for each HVAC design feature, we would not include other pathogens; however, for design features where we did not find studies specific to viruses, we would expand to other pathogens.* |  |
| HVAC | Design features relating to:   - Ventilation (ventilation rate, air changes per hour (ACH), air exchange, airflow pattern, pressurization) - Filtration (air filtration, filter type, MERV rating, filter age and/or use, pressure drop, holding capacity, replacement, change frequency) - Ultraviolet germicidal irradiation (UVGI; power, dose, uniformity of dose, flow rate, bioaerosol inactivation efficiency, location) - Humidity or relative humidity | Examines HVAC / mechanical / or other ventilation mechanisms overall, but not by specific design features. |
| Setting | - Office buildings - Public buildings (e.g., schools, day cares) - Residential buildings - Hospitals and other healthcare facilities (e.g., clinics) - Transport vehicles (e.g., aircraft) or hubs (e.g., airports) | - Outdoor settings - Indoor settings with natural ventilation |
| Outcomes | Quantitative data evaluating the correlation or association between virus transmission and above HVAC features | Qualitative data |
| Study design | Primary research, including:   - Epidemiological studies - Observational studies (e.g., cohort, case-control, cross-sectional) - Experimental studies (including human or animal) - Modelling studies, including CFD | - Review articles - Commentaries, opinion pieces - Qualitative studies |
| Language | English  *We planned a staged process where we would include studies in languages other than English if we do not identify English language studies for specific HVAC design features or if we identified clusters of potentially relevant studies in another language.* |  |
| Year | No restrictions |  |
| Publication status | Published, peer-reviewed | Unpublished, not peer-reviewed |

CFD = computational fluid dynamics; HVAC = heating, ventilation, and air conditioning; MERV = minimum efficiency reporting value; UVGI = ultraviolet germicidal irradiation
