# Supplementary material for: The impact of heating, ventilation and air conditioning (HVAC) design features on the transmission of viruses, including the 2019 novel coronavirus (COVID-19): A systematic review of humidity
Source: PLoS One. 2022 Oct 10;17(10):e0275654. doi: 10.1371/journal.pone.0275654 (PMC9550073; doi:10.1371/journal.pone.0275654)
Supplement: Attachment — Submitted filename: Humidity-HVAC SR PLOS-ONE Response to Reviewer 2022sept3.docx [file pone.0275654.s003.docx]

**Manuscript ID**: PONE-D-22-01074

**Title:** The impact of heating, ventilation and air conditioning (HVAC) design features on the transmission of viruses, including the 2019 novel coronavirus (COVID-19): a systematic review of humidity

Dear Dr. Shepherd,

Thank you for the opportunity to respond to your comments and the reviewer comments, and submit a revised version of our manuscript. We have provided a response to each comment below. We have uploaded a marked-up copy of our manuscript showing our changes, as well as an unmarked copy of the revised manuscript. We thank you for your time and consideration and look forward to your response.

Best Regards,

Brian Fleck Ph.D., P.Eng., ICD.D

Professor, Mechanical Engineering, UAlberta

**Journal requirements:**

When submitting your revision, we need you to address these additional requirements.

1. Please ensure that your manuscript meets PLOS ONE's style requirements, including those for file naming. The PLOSONE style templates can be found at
https://journals.plos.org/plosone/s/file?id=wjVg/PLOSOne_formatting_sample_main_body.pdf and https://journals.plos.org/plosone/s/file?id=ba62/PLOSOne_formatting_sample_title_authors_affiliations.pdf.

***Authors’ response:*** *We have reviewed the style template documents and made the required formatting changes.*

2. Thank you for stating the following in the Funding Section of your manuscript:

“This work is funded by a Canadian Institutes of Health Research (CIHR) Operating Grant: Canadian 2019 Novel Coronavirus (COVID-19) Rapid Research Funding Opportunity [https://webapps.cihr-irsc.gc.ca/decisions/p/project_details.html?applId=422567&lang=en]. Dr. Hartling is supported by a Canada Research Chair in Knowledge Synthesis and Translation. Drs. Fleck and Zhong are supported by the NSERC Discovery program.”

We note that you have provided funding information that is not currently declared in your Funding Statement. However, funding information should not appear in the Acknowledgments section or other areas of your manuscript. We will only publish funding information present in the Funding Statement section of the online submission form.

Please remove any funding-related text from the manuscript and let us know how you would like to update your Funding Statement. Currently, your Funding Statement reads as follows:

“This work is funded by a Canadian Institutes of Health Research (CIHR) Operating Grant, Canadian 2019 NovelCoronavirus (COVID-19) Rapid Research Funding Opportunity [https://webapps.cihr-irsc.gc.ca/decisions/p/project_details.html?applId=422567&lang=en], and Alberta Innovates. Funding was received by LZ. The funders played no role in study design, data collection and analysis, decision to publish, or preparation of the manuscript.”

Please include your amended statements within your cover letter; we will change the online submission form on your behalf.

***Authors’ response:*** *We would like the following funding statement to appear in the online submission; thank you for making this change on our behalf:* This work is funded by a Canadian Institutes of Health Research (CIHR) Operating Grant, Canadian 2019 Novel Coronavirus (COVID-19) Rapid Research Funding Opportunity [https://webapps.cihr-irsc.gc.ca/decisions/p/project_details.html?applId=422567&lang=en], and Alberta Innovates. Dr. Hartling is supported by a Canada Research Chair in Knowledge Synthesis and Translation. Drs. Fleck and Zhong are supported by the NSERC Discovery program. The funders played no role in study design, data collection and analysis, decision to publish, or preparation of the manuscript.

3. Thank you for stating the following in the Competing Interests section:

“BF is an unpaid advisor for Pura Air Inc. in Vancouver.”

Please confirm that this does not alter your adherence to all PLOS ONE policies on sharing data and materials, by including the following statement: ""This does not alter our adherence to PLOS ONE policies on sharing data and materials.” (as detailed online in our guide for authors http://journals.plos.org/plosone/s/competing-interests). If there are restrictions on sharing of data and/or materials, please state these. Please note that we cannot proceed with consideration of your article until this information has been declared.

Please include your updated Competing Interests statement in your cover letter; we will change the online submission form on your behalf.

***Authors’ response:*** *This is no longer current. BF is NOT an advisor for Pura Air Inc. We have removed this statement from the manuscript.*

4. Please upload a copy of Figures 3-5, to which you refer in your manuscript. If the figure is no longer to be included as part of the submission please remove all reference to it within the text.

***Authors’ response:*** *These figures were from studies that we included in our systematic review; we are citing the source of the data that we have included from these relevant studies. In Table 1 (Page 15) in the row labelled Noti 2013^16^, "Figure 3" and "Figure 4" refer to Figure 3 and Figure 4 in Noti 2013^16^. Similarly, in Table 2 (Page 22) in the row labelled Koep 2013^46^, "Figure 4" refers to Figure 4 in Koep 2013^46^. On Line 273-274, “To achieve 55kJ/kg specific enthalpy, HVAC settings require an indoor RH of approximately 93% at 20°C, decreasing almost linearly to 60%RH at 25°C (Fig. 5 [42]).”; “Fig. 5” refers to Figure 5 in Spena et al [42].*

*In the revised manuscript we have clarified, e.g., Figure 3 in Noti 2013, Figure 4 in Noti 2013, etc. Is this satisfactory? If not, we could change the figure numbers to the page numbers in the cited sources. See below:*

*Line 277*

*To achieve 55kJ/kg specific enthalpy, HVAC settings require an indoor RH of approximately 93% at 20°C, decreasing almost linearly to 60%RH at 25°C (Fig. 5 in Spena et al [42]).*

*Could change to: To achieve 55kJ/kg specific enthalpy, HVAC settings require an indoor RH of approximately 93% at 20°C, decreasing almost linearly to 60%RH at 25°C [42p10].*

*Table 1: Noti 2013*

*Increased RH from 43% to 73% associated with similar % infectivity (14.6% to ~17%; Figure 3 in Noti 2013 [16])*

*Could change to: Increased RH from 43% to 73% associated with similar % infectivity (14.6% to ~17%; p.5)*

*Increased RH from 20% to 45% associated with significant decrease in infectious virus (Figure 4 in Noti 2013 [16])*

*Could change to: Increased RH from 20% to 45% associated with significant decrease in infectious virus (p.6)*

*Table 2: Koep 2013*

*Increased RH from 40% to 60% associated with decreased influenza survival [~47% (Figure 4 in Koel 2013 [46]) to 34% survival (p.4)]*

*Could change to: Increased RH from 40% to 60% associated with decreased influenza survival [~47% (p.6) to 34% survival (p.4)]*

Please review your reference list to ensure that it is complete and correct. If you have cited papers that have been retracted, please include the rationale for doing so in the manuscript text, or remove these references and replace them with relevant current references. Any changes to the reference list should be mentioned in the rebuttal letter that accompanies your revised manuscript. If you need to cite a retracted article, indicate the article’s retracted status in the References list and also include a citation and full reference for the retraction notice.

***Authors’ response:*** *We have reviewed the reference list and made minor edits to ensure the references are complete and correct. There were no retractions.*

**Reviewers' comments:**

Reviewer #1:

General comments:

This study performs a systematic literature review on the effects of humidity on the viability inside HVAC systems for different viruses. The authors concluded that other parameters, including aerosol medium temperature and exposure time, influence the effect of humidity on virus viability and found that humidity effects on viability are not the same for all viruses, and consideration must be taken for each virus type. I believe the manuscript is well written and an important and useful topic for researchers interested in laboratory and field studies. Therefore, I accept the manuscript with a few changes:

1) You have identified low, mid, and high levels of RH, but instead of using these definitions on and off, I would consistently use the RH values.

***Authors’ response:*** *We recognize that we were inconsistent in the use of low, mid, and high RH in the text. We have corrected these inconsistencies throughout the manuscript (please see Tracked Changes). Now, we consistently use various forms of low RH, mid RH, high RH, low to mid RH, mid to high RH, and low to high RH. In addition, we added the humidity categorization in the beginning of the Results section on Line 188-190: “Details of individual studies are provided in tables and summarized in the sections that follow; humidity was categorized as low (<40%RH), mid (40-60%RH), and high (>60%RH) RH.” We feel that the use of low, mid, and high RH is easier for the reader to follow than repeating the RH range in every instance, and also allows for synthesis across studies that used slightly different values.*

2) Please correct the in-text citation when using an author's name. For example, in line 63, Derby et al., please add the reference number. Please review the manuscript and correct all similar instances.

***Authors’ response:*** *Where appropriate, we moved the reference number from the end of the sentence to appear after the author name for in-text citations throughout the manuscript (please see Tracked Changes). However, the example from Line 63 is different: “Derby et al reviewed the effect of low humidity (≤40% relative humidity [RH]) on virus viability and transmission [12] and identified several studies, both modelling and experimental, showing that humidity influenced virus transmission and virus survival [14-18].” Vancouver style indicates that “In-text citation numbers should be placed after the relevant part of a sentence.” (https://guides.hsict.library.utoronto.ca/vancouver-style). For the example from Line 63, different parts of the sentence refer to different citable information; therefore, the current citation formatting is retained.*

3) Please add two separate sections that address a) the limitations of this study, b) expand on "identify gaps in current research, which can guide future research priorities".

***Authors’ response:*** *We added a “Strengths and Limitations” section on Line 473-481 and a “Future Research and Practice Priorities” section on Line 482-491.*

Reviewer #2:

The authors systematically reviewed the effect of humidity on the survival and transmission of coronaviruses and influenza viruses. The work followed rigorous methods for study selection. It captured all the studies I would expect. The results are explained clearly, and the discussion effectively synthesizes the findings and relates them to the real world. I have just a few minor suggestions.

1. line 135: “A variety of agents were included in the search with priority placed on studies of viruses or agents that simulated viruses. Other agents (e.g., fungi, bacteria) would be included if studies were not available specific to viruses. Studies using bacteriophages, which are viruses that infect bacterial cells [26], were included.” This seems to contradict the objective stated in the introduction, which was to focus on “enveloped, ssRNA coronaviruses and influenza viruses as opposed to viruses more generally.”

***Authors’ response:*** *The following sentence has been added on Line 139: "For this specific review, the synthesis was further narrowed from viruses to coronaviruses and influenza viruses."*

2. line 232: “Influenza A/Mexico/4018/2019” I think this should be 2009 instead of 2019.

***Authors’ response:*** *You are correct. Thank you for catching this error. The text now reads “Influenza A/Mexico/4018/2009” on Line 235 and “A/Mexico/4018/2009” in Table 1 on Page 16.*

3. line 265: Consider pointing out that the modeling studies rely on experimental data, so their results are not independent of those presented in the previous subsection.

***Authors’ response:*** *We already carefully detail in the text the relationship between the modelling studies and the experimental data. Line 272-274 “Spena et al [42] used experimental data from Pyankov et al [38] and Van Doremalen et al [37] for MERS-CoV, MERS isolate HCov-EMC, SARS-CoV-1, and SARS-CoV-2 in the development of the model.” Line 285-287 “Three of the five studies [43,45,46] used animal transmission data from Lowen et al [14] and one study [17] included data from Harper [33] for aerosolized viruses.”*
